# Autoimmune Polyglandular Syndrome Type II

**DOI:** 10.7759/cureus.110735

**Published:** 2026-06-12

**Authors:** Pooja Gandhi, Pramod Gandhi, Shivani Solanki, Anaya Kamalapurkar, Raghuvanshi Ayush Atul

**Affiliations:** 1 General Medicine, N.K.P. Salve Institute of Medical Sciences and Research Centre and Lata Mangeshkar Hospital, Nagpur, IND; 2 Endocrinology and Diabetes, Gandhi Hospital, Nagpur, IND; 3 Medicine, N.K.P. Salve Institute of Medical Sciences and Research Centre and Lata Mangeshkar Hospital, Nagpur, IND

**Keywords:** addisonian crisis, autoimmune polyglandular syndrome type 2 (aps-2), hormone replacement therapy, hyperpigmentation, primary adrenal insufficiency, primary hypothyroidism, schmidt’s syndrome

## Abstract

Autoimmune Polyglandular Syndrome Type 2 (APS-2), also called Schmidt’s syndrome, is a rare endocrine disorder characterized by primary adrenal insufficiency with autoimmune thyroid disease or type 1 diabetes mellitus. We report a case of a 40-year-old female patient with a history of primary hypothyroidism who presented to the emergency department with abdominal pain, vomiting, hypotension, hypoglycemia, and progressive hyperpigmentation. Laboratory studies confirmed an acute Addisonian crisis and revealed profound hyponatremia, hypocalcemia, hypomagnesemia, low serum cortisol, high adrenocorticotropic hormone (ACTH), and an elevated thyroid-stimulating hormone (TSH). The physiologic stress of an incidental hormonally inactive left ovarian serous cystadenoma likely precipitated this acute life-threatening event and also contributed to the unmasking of the patient’s underlying adrenal deficiency. She was successfully resuscitated with intravenous fluids, vasopressors, and corticosteroid replacement and was discharged on a long-term regimen of oral steroids and thyroxine. This case highlights the significance of a high clinical suspicion for concomitant endocrine failures in patients with known autoimmune endocrinopathies presenting with acute systemic deterioration, as an early multidisciplinary intervention is key to improving prognosis and avoiding death.

## Introduction

Autoimmune Polyglandular Syndrome Type 2 (APS-2) is often called Schmidt’s syndrome and most often affects women in early to mid-adulthood [1]. The disease is characterized by autoimmune destruction of various endocrine glands, particularly the adrenal cortex and thyroid. People come in with generalized, vague symptoms. Finally, the underlying pathology declares itself in an acute adrenal crisis [2]. Adrenal crisis is a life-threatening endocrine emergency characterized by hypotension, hypoglycemia, and electrolyte disturbances due to acute cortisol deficiency. We report this case to highlight a clinically likely APS‑2 presenting with an Addisonian crisis in a woman with longstanding hypothyroidism and the presence of a large ovarian serous cystadenoma and unrecognized multi‑gland involvement leading to delayed recognition.

## Case presentation

**Figure 1:**
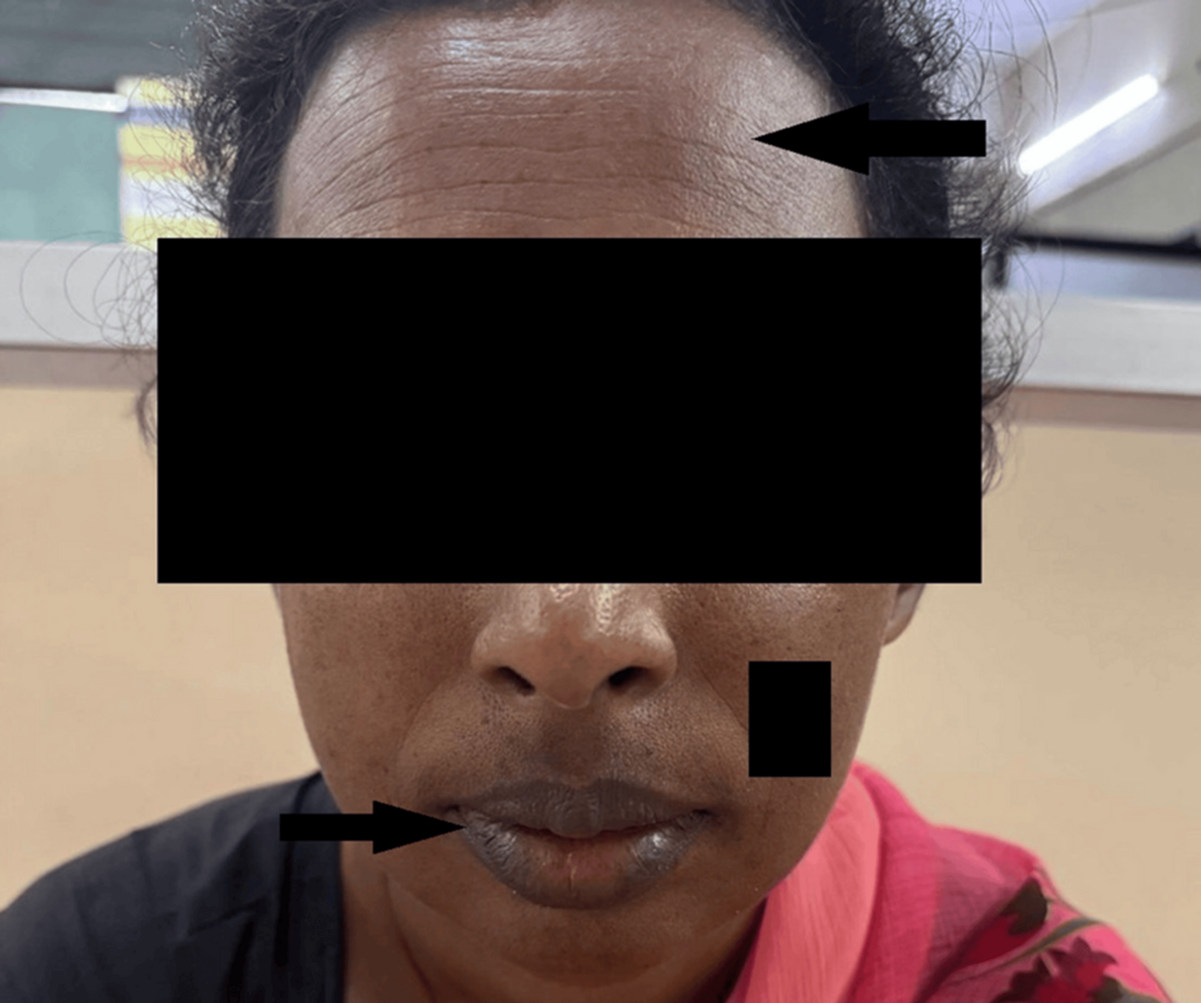
Clinical photograph of the patient's face The image demonstrates diffuse skin hyperpigmentation, particularly noticeable on the forehead and perioral region, characteristic of primary adrenal insufficiency.

**Figure 2:**
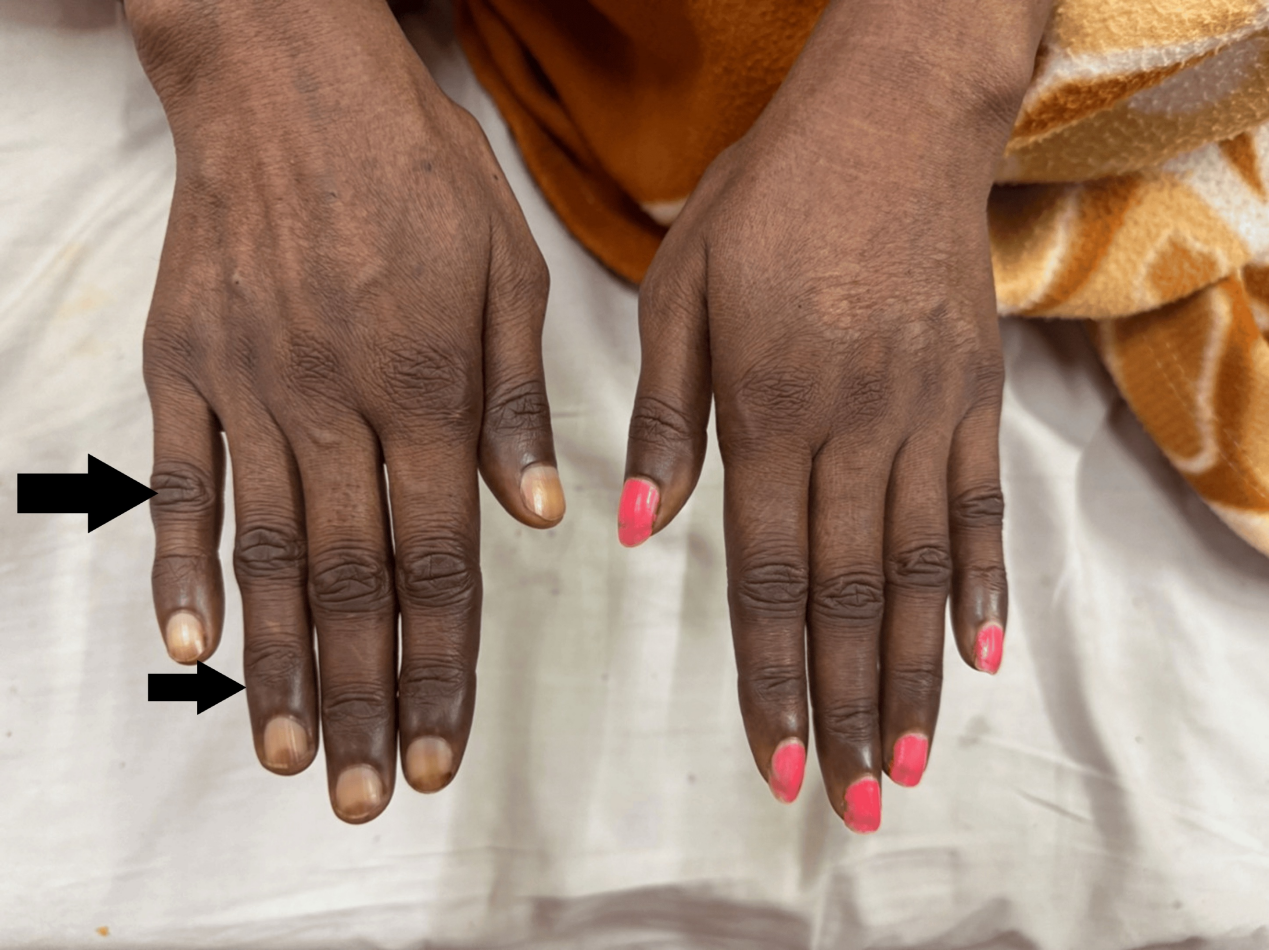
Clinical photograph of the patient's hands The image displays pronounced hyperpigmentation on the dorsal surfaces, with marked darkening accentuated over the metacarpophalangeal and interphalangeal joints (knuckles).

A 40-year-old woman presented to the emergency department in N.K.P. Salve Institute of Medical Sciences and Research Centre and Lata Mangeshkar Hospital, Nagpur, India, with abdominal pain for four days and vomiting for one day. Her past medical history was significant for a one-year history of primary hypothyroidism on 50 mcg of levothyroxine daily. She had persistent localized abdominal pain. Her emesis was clear fluid without projectile. Further questioning revealed a history of several previous episodes of hypotension over the last six to eight months, treated symptomatically by a local practitioner. She reports progressive darkening of the skin over her face, hands, and generalized body over the past several months, becoming more apparent in the weeks prior to presentation (Figures 1, 2).

The patient was initially admitted under the surgical service for a large pelvic mass seen on ultrasound. She was then transferred to the medical intensive care unit for continued care for low blood pressure and ongoing hypoglycemia. Initial laboratory studies showed a plasma glucose of 45 mg/dL, consistent with hypoglycemia in the setting of hypotension and systemic illness. The physical exam was consistent with tachycardia, pallor, and the hyperpigmentation described above.

Laboratory investigations revealed hyponatremia, mild hyperkalemia, hypocalcemia, and hypomagnesemia, with a serum potassium of 5.6 mEq/L, supporting primary adrenal insufficiency (Table 1). A low serum cortisol level (0.54 mcg/dL) in the early morning, together with a very high ACTH (273 pg/mL), confirmed primary adrenal insufficiency. Serum albumin was 3.8 g/dL, and the albumin‑corrected calcium was 8.1 mg/dL, confirming true mild hypocalcemia. Intact parathyroid hormone (PTH) was 65 pg/mL, and 25‑OH vitamin D was mildly reduced at 18 ng/mL, suggesting vitamin D insufficiency with an appropriate parathyroid hormone (PTH) response. In addition, she had an elevated TSH (24.06 µIU/mL), consistent with her pre-existing thyroid disease.

**Table 1:** Laboratory investigations results on admission ACTH: Adrenocorticotropic Hormone, TSH: Thyroid Stimulating Hormone, PTH: Parathyroid Hormone.

Parameter	Patient Value	Reference Range / Normal Values	Interpretation
Serum Sodium	128 mEq/L	135 – 145 mEq/L	Hyponatremia
Serum Potassium	5.6 mEq/L	3.5 – 5.0 mEq/L	Mild hyperkalemia
Serum Calcium	7.9 mg/dL	8.5 – 10.5 mg/dL	Low total calcium
Serum Albumin	3.8 g/dL	3.5 – 5.0 g/dL	Normal
Corrected Calcium	8.1 mg/dL	8.5 – 10.5 mg/dL	Mild hypocalcemia
Serum Magnesium	1.1 mg/dL	1.7 – 2.2 mg/dL	Hypomagnesemia
Serum Glucose	45 mg/dL	70 – 140 mg/dL	Hypoglycemia
Serum Cortisol (8:00 AM)	0.54 mcg/dL	5.0 – 25.0 mcg/dL	Critically low
ACTH	273 pg/mL	10 – 60 pg/mL	Markedly elevated
TSH	24.06 µIU/mL	0.4 – 4.0 µIU/mL	Elevated
Intact PTH	65 pg/mL	15 – 65 pg/mL	High‑normal
25‑OH Vitamin D	18 ng/mL	20 – 50 ng/mL	Mild deficiency

Imaging of the pelvis was suggestive of a left ovarian serous cystadenoma. The presence of both primary adrenal insufficiency and primary hypothyroidism in this patient is highly suggestive of autoimmune polyglandular syndrome type 2 (APS-2), but serologic confirmation with adrenal (21-hydroxylase) and thyroid autoantibodies was not obtained and remains a limitation of this case. Interventions included intravenous fluids, vasopressors, and corticosteroid replacement with hydrocortisone and fludrocortisone. She was discharged on oral steroids and thyroxine with strict instructions for outpatient monitoring of the ovarian cyst after clinical improvement and stabilization of vital signs.

## Discussion

APS-2 is a rare endocrine disease, defined by primary adrenal insufficiency associated with thyroid autoimmunity or type 1 diabetes. The clinical phenotype and hormone profile of our patient are compatible with this constellation, but we did not perform 21-hydroxylase, adrenal cortex, anti-TPO, or anti-thyroglobulin antibody testing, so autoimmunity cannot be serologically proven. Thus, we term this a clinically probable APS-2 and emphasize the importance of extensive autoantibody testing in similar cases. It is the most frequent polyglandular autoimmune syndrome in adults, and it predominates in women in the third and fourth decades of life [3,4]. Polygenic etiologies of the disease are closely linked with human leukocyte antigen (HLA)-DR3 and HLA-DR4 haplotypes [5].

Our case had known hypothyroidism with an acute Addisonian crisis presenting with hypotension, hypoglycemia, gastrointestinal symptoms, hyperpigmentation, and electrolyte disturbance. Normocalcemia or hypercalcemia is commonly seen in primary adrenal insufficiency. This patient had biochemical hypocalcemia. Corrected for albumin, calcium was still mildly low, with a high-normal PTH and mild vitamin D deficiency, and no clinical features of autoimmune hypoparathyroidism or APS-1 (e.g., chronic mucocutaneous candidiasis or ectodermal dystrophy). We therefore attribute the hypocalcemia to a combination of acute critical illness, concomitant hypomagnesemia, and vitamin D insufficiency rather than a separate autoimmune parathyroid process. Adrenal failure was confirmed by specific clinical signs and conclusive hormonal assays. The association of two different endocrinopathies met the criteria for the diagnosis of APS-2 [6]. Many people develop autoimmune problems as they age.

The most dangerous manifestation is the Addisonian crisis, which is often induced by physical stress, infection, and surgical procedures [7]. In our case, physiologic stress from a large serous ovarian cystadenoma most likely precipitated the acute event. Although not directly related to APS-2, it was hormonally inactive, but was sufficiently stressful metabolically to bring her underlying adrenal deficiency to light.

The initiation or escalation of thyroxine therapy in the context of undiagnosed adrenal insufficiency can precipitate an adrenal crisis due to increased cortisol clearance and higher metabolic demands, and this is an important general teaching point. In our patient, however, the levothyroxine dose (50 mcg daily) had been stable for approximately one year, with no recent initiation or dose increase, making this mechanism unlikely to have precipitated the acute crisis [8]. In our case, physiologic stress from a large serous ovarian cystadenoma most likely precipitated the acute event. Although not directly related to APS-2, it was hormonally inactive, but was sufficiently stressful metabolically to bring her underlying adrenal deficiency to light.

Long-term management involves lifelong hormone replacement therapy with glucocorticoids (hydrocortisone) and mineralocorticoids (fludrocortisone) and ongoing thyroxine replacement. Long-term safety includes patient education on “sick-day rules,” the use of stress-dose steroids during illness, and early warning signs [9]. Routine surveillance for associated autoimmune conditions-such as type 1 diabetes, celiac disease, pernicious anemia, and premature ovarian failure-is highly recommended due to the polyendocrine nature of the illness [10]. This scenario ultimately highlights the importance of a high clinical suspicion in patients presenting with an acute systemic deterioration with known autoimmune endocrinopathy. A multidisciplinary approach to care and prompt intervention are critical to improving prognosis and overall health.

## Conclusions

APS-2 is frequently diagnosed late and can manifest as a life-threatening Addisonian crisis. This case highlights the importance of early recognition and management of multiple endocrine failures in autoimmune settings. In the future, clinicians should keep a high index of suspicion for patients with pre-existing endocrine conditions who present with nonspecific, worsening, or general symptoms. A comprehensive patient education program that includes medication adherence, the physiologic effects of sudden physical stress, and the importance of emergency protocols can be the cornerstone for preventing acute decompensation. Ultimately, the establishment of robust interdisciplinary dialogue between endocrinologists, emergency physicians, and primary care providers will ensure cohesive, proactive, and life-saving management for this vulnerable patient population.
